# The Response of Murine Gut Microbiome in the Presence of Altered *rpoS* Gene of *Klebsiella pneumoniae*

**DOI:** 10.3390/ijms25179222

**Published:** 2024-08-25

**Authors:** Muhammad Zafar Iqbal, Pengfei He, Pengbo He, Yixin Wu, Shahzad Munir, Yueqiu He

**Affiliations:** State Key Laboratory for Conservation and Utilization of Bioresources in Yunnan, Yunnan Agricultural University, Kunming 650201, China

**Keywords:** gut, microbiome, cross kingdom, *rpoS*, infection

## Abstract

The murine model is invaluable for studying intricate interactions among gut microbes; hosts; and diseases. However; the impact of genetic variations in the murine microbiome; especially in disease contexts such as *Klebsiella pneumoniae* (*Kp*) infection; still needs to be explored. *Kp*; an opportunistic global pathogen; is becoming increasingly prevalent in regions like Asia; especially China. This study explored the role of the gut microbiota during *Kp* infection using mouse model; including wild-type and *rpoS* mutants of Kp138; KpC4; and KpE4 from human; maize; and ditch water; respectively. Under stress conditions; RpoS reconfigures global gene expression in bacteria; shifting the cells from active growth to survival mode. Our study examined notable differences in microbiome composition; finding that *Lactobacillus* and *Klebsiella* (particularly in WKp138) were the most abundant genera in mice guts at the genus level in all wild-type treated mice. In contrast; *Firmicutes* were predominant in the healthy control mice. Furthermore; *Clostridium* was the dominant genus in all mutants; mainly in ∆KpC4; and was absent in wild-type treated mice. Differential abundance analysis identified that these candidate taxa potentially influence disease progression and pathogen virulence. Functional prediction analysis showed that most bacterial groups were functionally involved in biosynthesis; precursor metabolites; degradation; energy generation; and metabolic cluster formation. These findings challenge the conventional understanding and highlight the need for nuanced interpretations in murine studies. Additionally; this study sheds light on microbiome–immune interactions in *K. pneumoniae* infection and proposes new potential therapeutic strategies.

## 1. Introduction

The gut microbiota substantially impacts the gut in sustaining the host health. Infectious interruptions to the balanced gut microbiome have been associated with inflammatory, metabolic, and autoimmune diseases and can even lead to cancer, mental illness, and developmental disorders [1,2]. In recent years, numerous studies have been conducted to investigate which factors impact the gut microbiota, such as antibiotics, diet, health, nutrition, and age [3]. *Klebsiella pneumoniae* is resistant to nearly all known antibiotics used for treatment and therefore poses the most significant and severe risks to human health. Drug-resistant strains of *K. pneumoniae* are associated with extremely high lethality rates, where bloodstream and other multiple infections often result in mortality rates of 50% [4]. In this regard, it is a significant challenge to determine an effective clinical treatment. A clinical study found that 22.8% of *K. pneumoniae* isolates were related to invasive infections [5], potentially indicating the upcoming crisis and challenges in clinical infection therapeutics [6]. As a result, this emphasizes the significance of elucidating the precise relationships and mechanisms of virulence factors and antimicrobial resistance in virulent pathogenic *Klebsiella* isolates.

Research on gut microbiomes is dominated by murine models due to their low costs, higher rate of reproduction, relativity close resemblance to the human gut system, and ease of experimental manipulation. The disturbance in the microbiome due to pathogen intervention requires investigating the possible cause and relationships between dysbiosis and disease outcomes. Cross-study comparisons can be complicated due to various factors that affect the diversity of the mouse gut microbiome, especially when the alterations in the *rpoS* gene are infrequent [7,8].

*RpoS* is a crucial stress response regulator, serving as an alternate sigma factor of RNA polymerase, primarily present in *Gammaproteobacteria* [9,10]. In various pathogens, *RpoS* is reported to play a crucial role in cell survival under stressful conditions, such as oxidative stress and exposure to acids [11,12,13]. However, its function in the pathogenesis and impact on virulence may vary, even within the same species [11,12,13,14]. Under harsh conditions, *RpoS* modifies the comprehensive gene expression of bacteria, causing the cells to shift from an active growth state to the survival stage [15]. Additionally, it has also been demonstrated to promote bacterial colonization in the gut. For instance, *RpoS* is necessary for various pathogenic bacterial species to effectively colonize the intestine, such as *Salmonella enterica* serovar Typhimurium, *Vibrio cholerae*, and the highly pathogenic *Escherichia. coli* [16,17]. To explore bacterial challenges, we investigated the role of *RpoS* in gut colonization in mice. The results showed that the absence of *RpoS* reduces *Klebsiella*’s capacity to colonize the mouse gut, while this pattern was not followed in wild-type *Kp* strains (Kp138, KpE4, and KpC4).

This study was designed to compare the impact of the *RpoS*-mutant and wild-type (WT) effects on microbiome variability within the framework of a disease progression in a mouse model. We investigated how comparative effects influence the variability in the gut microbiome of albino laboratory mice, and we found notable differences in the microbiome composition based on the mutation. The microbiome variations in the wild-type (WT) and Mutant-treated mice were not identical. However, differential abundance analysis identified several operational taxonomic units and conserved candidate taxa that could be involved in developing progression or reducing illness.

Our research aimed to explore the microbial diversity of healthy and diseased mice, examining the differences in the relative abundance, phylogenetic diversity, and species richness of the microbial communities in symptomatic mice inoculated with the *Klebsiella* pathogen. Our findings indicated that infecting mice with *K. pneumoniae* significantly altered the healthy gut microbiota and that induced gut illness resulted in evident changes in certain bacterial species. Understanding bacterial colonization can help scientists study how bacteria interact with the gut and develop new strategies to prevent harmful bacteria from colonizing or causing infection.

## 2. Results

### 2.1. Most Dominant Taxa in Mice in the Presence of Wild-Type and Mutant of K. pneumoniae

**Figure 1:**
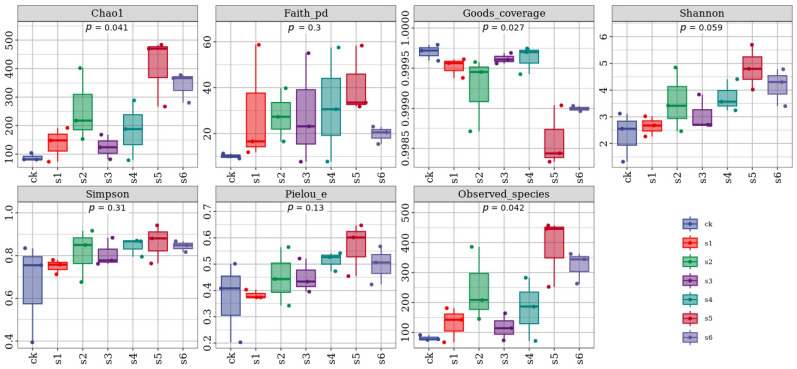
Alpha diversity analysis of the treatments. In this context, coordinates represent the categories used for grouping, while the ordinate denotes the index values of alpha diversity. The numbers below the diversity index labels indicate the *p*-values derived from Kruskal–Wallis test. CK: control. S1: WT Kp138. S2: ∆Kp138. S3: WT KpC4. S4: ∆KpC4. S5: WT KpE4. S6: ∆KpE4.

The bacterial microbiome from the gut of healthy, mutant, and wild-type *K. pneumoniae*-treated mice was sequenced. The QIIME cutadapt tool was used for pair-end reads. The excision sequence was selected through the primer fragment, and the mismatched primers were removed. The QIIME DADA2 denoised function for paired-end data was used for quality control by the DADA2 algorithm, and the following steps of de-dimerization, denoising splicing, and chimerization removal were performed. The box plot results are shown in Figure 1. The one-way analysis of variance (ANOVA) results showed significantly different (*p* < 0.001) alpha diversity indices across all strains of *K. pneumoniae*, i.e., healthy, wild, and mutant. Alpha diversity analysis was carried out to examine the evenness and richness within a single microbial ecosystem of species.

**Figure 2:**
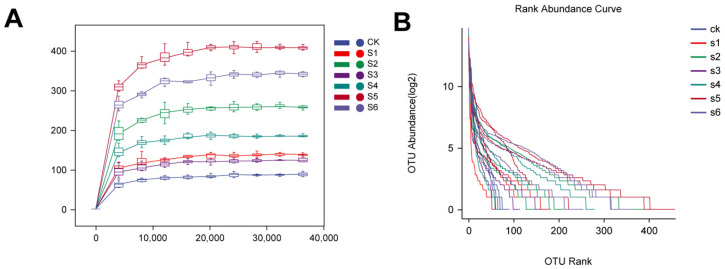
Sparse curve and rank abundance curve. (**A**) Sample sparsity curves show the degree of leveling on the x-axis and the median value of the alpha diversity index, which is calculated ten times and plotted as a box plot on the y-axis. CK: control. S1: WT Kp138. S2: ∆Kp138. S3: WT KpC4. S4: ∆KpC4. S5: WT KpE4. S6: ∆KpE4. (**B**) The sample abundance rank curve plots ASV serial numbers by their abundance on the x-axis. At the same time, the y-axis depicts the abundance values of ASVs within the sample or group after the Log2 transformation. CK: control. S1: WT Kp138. S2: ∆Kp138. S3: WT KpC4. S4: ∆KpC4. S5: WT KpE4. S6: ∆KpE4.

Seven samples of healthy and diseased mice with three replications were used to check alpha diversity. The results showed that alpha diversity indexes of OTU level with Chao, Shannon, Simpson, observed species, Faith, Pielou, and Goods coverage or gut samples of healthy mice showed higher values compared to diseased mice. In brief, the alpha diversity of healthy mice samples significantly differs from that of diseased mice. The Shannon (4.68), Simpson (0.05), Faith (704.77), and Chao (717.33) indices of healthy mice show significant differences from those of diseased mice. The rarefaction curves of all samples nearly hit the saturation peak (Figure 2A), showing that the sequenced findings adequately reflect the variety present in the samples. The quantity of new unknown amplicon sequence variations (ASVs) did not rise as the sequence depth increased. The SPN and SPD were equal and homogeneous, but the SNPN and SOPD exhibited a considerable variance in abundances across OTUs and poor homogeneity.

### 2.2. Dominant Microbiota in the Healthy Mice

**Figure 3:**
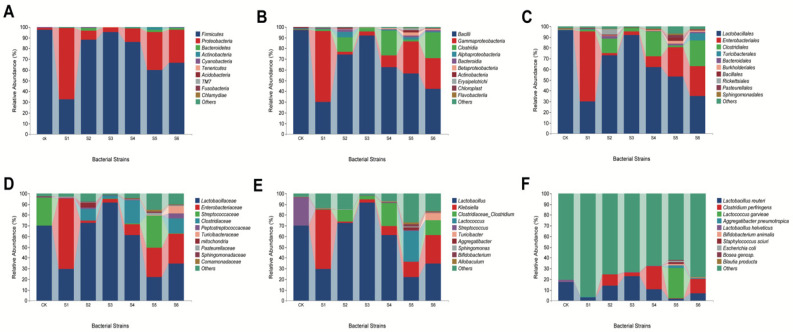
Relative abundance of bacterial communities (**A**) Distribution of microbial community abundances at the phylum level in all samples of *K. pneumoniae*-infected mice gut. (**B**) Abundance proportions of microbial communities at the class level across all samples. (**C**) Proportional abundances of bacterial communities at the order level across all samples. (**D**) The family-level proportional abundances of microbial communities across all samples. (**E**) Proportions of microbial community abundances at the genus level in all samples. (**F**) The proportions of microbial communities at the species level across all samples. CK: Control. S1: WT Kp138. S2: ∆Kp138. S3: WT KpC4. S4: ∆KpC4. S5: WT KpE4. S6: ∆KpE4.

The abundance of bacterial community at the phylum level in the gut microbiome was observed in healthy control albino mice. After one week of the experiment, Firmicutes showed the maximum comparative abundance of 97.43%, followed by Proteobacteria (1.52%). However, maximum values were observed at the class level for *Bacilli*, *Gammaproteobacteria*, and *Clostridia* (96.97%, 0.36%, and 0.46%). Furthermore, at the order level, maximum comparative abundance was observed for *Lactobacillales* (96.59%), while *Enterobacteriales* were absent in abundance. *Lactobacillaceae* (70.04%) and the absence of *Enterobacteriaceae* were observed, whereas *Streptococcaceae* was only found (26.23%) at the family level. *Lactobacillus* (70.02%) and *Klebsiella* were absent, while *Streptococcus* was only 26.14% in the control at the genus level. However, at the species level, the maximum relative abundance of *Lactobacillus reuteri* in the control (17.33%) was observed (Figure 3A–F).

### 2.3. Presence of Dominant Microbiota in WT Kp-Treated Mice

After one week of the application, maximum comparative abundance was observed for Firmicutes (95.46%), followed by Proteobacteria (4.13%) with WT *K. pneumoniae* KpC4 application. Similarly, WT KpE4 accounted for 59.81% of Firmicutes and 35.39% of Proteobacteria, and for Kp138, it was 32.41% and 66.83%, respectively. It was also observed that *Bacteroidetes* and *Actinobacteria* were found in WT KpE4, accounting for 2.20% and 2.17%, respectively. At the class level, *Bacilli*, *Gammaproteobacteria*, and *Clostridia* were observed in WT KpC4 (91.98%, 3.61%, and 3.48%, respectively), WT KpE4 (56.46%, 30.13%, and 1.51%, respectively), WT Kp138 (30.22%, 66.10%, and 1.67%, respectively). *Gammaprotobacteria* abundance was found to be the highest in WT Kp138 (66.10%), whereas the lowest abundance was recorded for actinobacteria and *Betaprotobacteria* in WT KpE4 (2.13% and 2.69%, respectively). At the order level, a maximum abundance of *Lactobacillales* and *Enterobacteriales* order was observed in WT KpC4 (91.90% and 3.34%, respectively), WT KpE4 (53.31% and 27.31%, respectively), and WT Kp138 (29.95% and 65.55%, respectively). At the family level, maximum comparative abundance was recorded for *Lactobacillaceae* in WT KpC4 (91.47%), whereas the minimum was observed in WT KpE4 (22.11%). Similarly, the *Enterobacteriaceae* family was observed in WT Kp138 in the range of 65.55%. Furthermore, *Streptococcaceae* was exclusively detected in WT KpE4 (29.84%) and the control group. At the genus level, *Lactobacillus* abundance was 91.45% in WT KpC4, while a minimum of 22.11% was found in WT KpE4. The maximum relative abundance of *Klebsiella* was observed in the WT138 (55.25%) group. However, the species level showed a maximum relative abundance of *Lactobacillus reuteri* in WT KpC4 (22.89%) and a minimum in WT KpE4 (2.01%). Meanwhile, the relative abundance of *Lactococcus garvieae* was found in a range of 28.53% only in WT KpE4, as illustrated in Figure 3A–F.

### 2.4. Role of Microbiota in the Mutant Treated Mice

**Figure 4:**
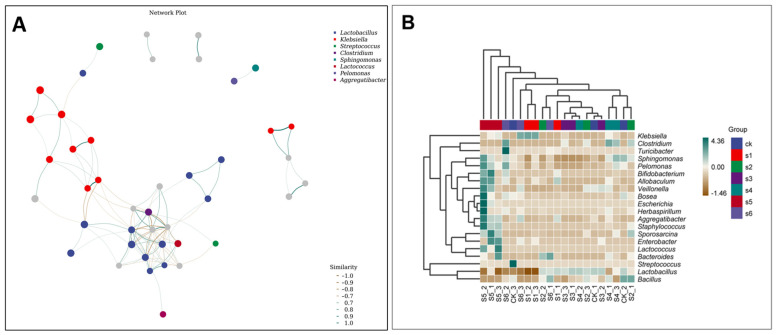
Association networks and species composition heat map. (**A**) The nodes in the visualization represent ASVs present in the sample, where the size of each node scales proportionally to its abundance (measured in log2 (CPM/n)). The visualization also identifies the top 10 nodes (modules) using distinct colors. Lines connecting nodes represent correlations between pairs of connected nodes. (**B**) The species composition heat map displays genus-level abundances, sorted by their highest concentrations, and features the top 20 genera. Using UPGMA clustering based on Euclidean distances derived from species composition data, the genera are grouped based on clustering results. On the y-axis, the species are grouped with UPGMA and organized according to their Pearson correlation coefficient matrix from composition data. CK: control. S1: WT Kp138. S2: ∆Kp138. S3: WT KpC4. S4: ∆KpC4. S5: WT KpE4. S6: ∆KpE4.

The relative abundance of the microbial community composition at the phyla level in the gut microbiome was observed in albino mice infected by mutant strains of *K. pneumoniae* (∆Kp138, ∆KpE4, and ∆KpC4). The maximum comparative abundance of Firmicutes and Proteobacteria phylum was observed with the application of mutant-type ∆KpC4 (85.91% and 13.02), ∆KpE4 (66.72% and 30.81%), and ∆Kp138 (88.23% and 8.28%), respectively. It was also observed that the maximum *Bacteroidetes* phylum was present in ∆Kp138 (2.44%). At the class level, the gut microbiome showed a maximum comparative abundance of *Bacilli*, *Gammaproteobacteria*, and *Clostridia* in ∆KpC4 (62.52%, 11.00%, and 23.20%, respectively). Similarly, *Bacilli, Gammaproteobacteria*, and *Clostridia* were present in ∆KpE4- (42.28%, 28.67%, and 23.95%) and ∆Kp138 (74.25%, 2.39%, and 13.69%)-treated mice. It was also observed that the maximum *Alphaproteobacteria* class was present in ∆Kp138 (5.19%). At the order level, the relative abundance of *Lactobacillales* and *Enterobacteriales* was observed in ∆KpC4 (62.06% and 10.15%, respectively), ∆KpE4 (35.26% and 27.63%, respectively), and ∆Kp138 (73.17% and 1.88%, respectively). *Clostridiales* abundance was the maximum in ∆KpE4 (23.95%), whereas *Turicibacterales* abundance was 6.72% exclusively in ∆KpE4. Similarly, *Rickettsiales* was only present in ∆Kp138 (4.75%). At the family level, the application of ∆Kp138 resulted in the lowest taxa of *Enterobacteriaceae* (1.88%). Additionally, *Turicibacteraceae* was mainly found in ∆KpE4, comprising 6.72% of the total observed taxa. At the genus level, a maximum abundance of *Klebsiella* (1.26%) was found in ∆Kp138. However, the species level showed a maximum relative abundance of *Clostridium perfringens* in ∆KpC4 (21.46%), as shown in Figure 3A–F. The Spearman’s rank correlation between *Lactobacillus* and *Klebsiella* was positive, suggesting a strong association between these genera. Nodes represent the ASV/OTU in the sample, and the node size is proportional to its abundance (in Log2 (CPM/n)), while the module with the most nodes in the top 10 nodes is identified by different colors (Figure 4A). The 20 most predominant with the highest correlations bacterial networks are shown in (Figure 4B). The first column is the name of the taxon, and the second to the last column is the relative abundance of the corresponding taxa in each sample of the grouping scheme. The samples are grouped according to the Euclidean distance between their species compositions.

### 2.5. Microbial Communities’ Comparisons in Healthy, Wild and Mutant Mice

The WT-treated mice guts exhibited a greater microbial abundance of *Klebsiella* than those of the mutant and healthy mice guts. Very few variations among the bacterial communities of all treated mice were observed. The top abundant genera were *Lactobacillus*, *Klebsiella*, and other bacteria (Figure 3). The gut microbiome of albino mice affected by *K. pneumoniae* displayed notable changes in microbial community composition at the phyla level. Firmicutes and Proteobacteria were most abundant, particularly in WT KpC4 (95.46% and 4.13%) compared to ∆KpC4 (85.91% and 13.02%). *Bacteroidetes* predominated in ∆Kp138 (2.44%), while in WT KpE4, *Bacteroidetes* and *Actinobacteria* were present at 2.20% and 2.17%, respectively. At the class level, *Bacilli*, *Gammaproteobacteria*, and *Clostridia* were prominent. WT KpC4 exhibited the highest levels (91.98%, 3.61%, and 3.48%) compared to mutants.

*Alphaproteobacteria* was notably elevated in ∆Kp138 (5.19%), while *Actinobacteria* and *Betaproteobacteria* were prominent in WT KpE4 at 2.13% and 2.69%, respectively. Analysis at the order level revealed a high abundance of *Lactobacillales* and *Enterobacteriales*, particularly in WT KpC4 (91.90% and 3.34%). WT KpE4 displayed dominance of *Clostridiales*, especially in mutants (23.95%), while *Turicibacterales* were exclusive to ∆KpE4 (6.72%), and *Rickettsiales* only present in ∆Kp138 (4.75%). Family-level analysis highlighted *Lactobacillaceae* predominance in WTC4 (91.47%) and minimal presence in WT KpE4 (22.11%).

*Enterobacteriaceae* were notably abundant in WT Kp138 (65.55%) compared to mutant treatments. *Streptococcaceae* were found in control and WT KpE4 (26.23% and 29.84%, respectively), while *Turicibacteraceae* were exclusive to ∆KpE4 (6.72%). At the genus level, *Lactobacillus* was highly abundant in WT KpC4 (91.45%) and the least in WT KpE4 (22.11%). *Klebsiella* was predominant in WT Kp138 (55.25%) compared to mutants. *Streptococcus* was only present in the control group. Further analysis at the species level revealed *Lactobacillus reuteri* dominance in WT KpC4 (22.89%), and *Lactococcus garvieae* was exclusively present in WT KpE4 (28.53%) (Figure 3A–F).

A community composition study was conducted across the samples to identify more species groups and their comparisons using heat maps, with the 20 most abundant genera chosen (Figure 4B). The categorization hierarchy tree depicts the hierarchical connections between all taxa in the sample population, ranging from phylum to genus. The node size is proportional to the average relative abundance of the taxa. The top 20 taxa by relative abundance are also designated by letters in the picture, with the shadow color matching the color of the relevant node. Samples were first grouped based on their components’ similarity and then sorted horizontally and vertically by the clustering findings. Similarly, the classification unit was grouped according to the degree of similarity spread across different samples and placed vertically based on the cluster findings.

**Figure 5:**
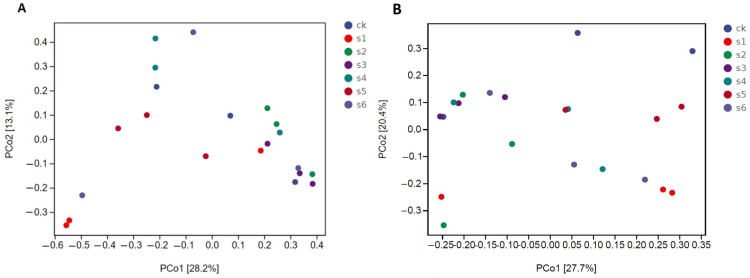
PCoA analysis. (**A**) Principal component analysis (PCoA) visualizing bacterial community variations among different tissues, utilizing Bray–Curtis distances. (**B**) Principal component analysis (PCoA) illustrating bacterial community distinctions across various tissues, employing Weighted UniFrac distances. CK: control. S1: WT Kp138. S2: ∆Kp138. S3: WT KpC4. S4: ∆KpC4. S5: WT KpE4. S6: ∆KpE4.

We used principal coordinate analysis (PCoA) based on the Bray–Curtis distance and weighted UniFrac distance to analyze the community similarity among samples. The PCoA scatter plot highlights the two characteristics of sample coordinates that accounted for the most significant differences, with contribution impacts of 67.4 and 11%, respectively, depending on the Bray–Curtis distance. This indicated that the gut bacterial community is different in WT compared to mutant and healthy mice (Figure 5B).

A Venn diagram was utilized to identify common and distinct OTUs across diverse variables, such as sample type. This graphic illustration depicted the overlap of operational taxonomic units (ASVs) between the WT and *rpoS* mutant bacteria in mice (Figure 5A). In the figure, each color block represents a group, the overlapping area between the color blocks indicates the ASV/OTU shared by the corresponding groups, and the number of each block indicates the number of ASV/OTU contained in the block. Specifically, 21 shared ASVs were observed, comprising the bacteria in the mice’s gut. According to the Venn diagram, out of 2239 OTUs, total mice samples showed SCK (101), S1 (144), S2 (341), S3 (106), S4 (224), S5 (701), and S6 (622) OTU levels.

### 2.6. Functional Predictions of Bacterial Taxa in Healthy, Wild, and Mutant-Treated Mice

**Figure 6:**
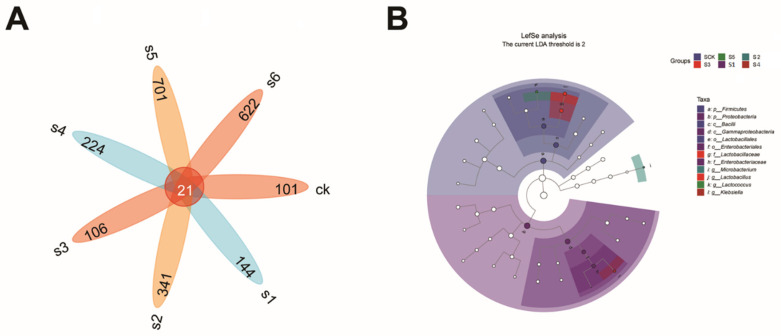
ASV/OTU Venn diagram and LEfSe analysis. (**A**) Venn diagram of bacterial community ASVs in the gut of mice infected with *Klebsiella*. CK: control. S1: WT Kp138. S2: ∆Kp138. S3: WT KpC4. S4: ∆KpC4. S5: WT KpE4. S6: ∆KpE4. (**B**) Bacterial communities were identified with a linear discriminant examination (LDA) score exceeding 2 across all gut samples. The taxonomic branching plots depict hierarchical relationships of the main taxa within a sample community, arranged from phylum to genus level (from inner to outer circle). Sizes of nodes reflect the average relative microbial abundance of each taxon. Hollow nodes indicate taxa without significant differences between groups, while colored nodes (e.g., green and red) denote taxa showing significant differences between groups. Letters identify taxa with significant differences from each other.

Clustering was used to distinguish between high- and low-abundance taxa, with color gradients representing the similarity in community composition throughout the samples. The results from hierarchical clustering showed that *Lactobacillus* and Protobacteria were the most abundant taxa at the genus level in the mice gut (Figure 6A). Compared to the respective mutants, wild strains (WT Kp138 and WT KpC4) exhibited a higher abundance of these bacteria when compared to the control group. Mutant ∆KpE4 also exhibited a significant presence of Protobacteria, whereas *Clostridium* was predominant in mutant ∆KpC4. Hence, *Clostridium* has emerged as the second most abundant bacteria after *Klebsiella*.

To examine the significant and notable differences in biomarkers among microbial communities and to distinguish genera across groups, linear discriminant analysis effect size (LEfSe) was used as a standard (LDA) value > 2 (Figure 6B). The control or healthy group exhibited the most remarkable bacterial taxonomic diversity (LDA > 2), featuring three genera primarily from *Lactobacillus* and Firmicutes (*Bacilli*). The WT KpC4 group had the second-highest diversity, featuring two genera primarily from *Lactobacillaceae* and *Lactobacillus*. The WT KpE4 group showed the lowest diversity (LDA > 2), with only one genus (*Lactococcus*). LEfSe was also utilized to elucidate significant biomarker differences across the samples.

**Figure 7:**
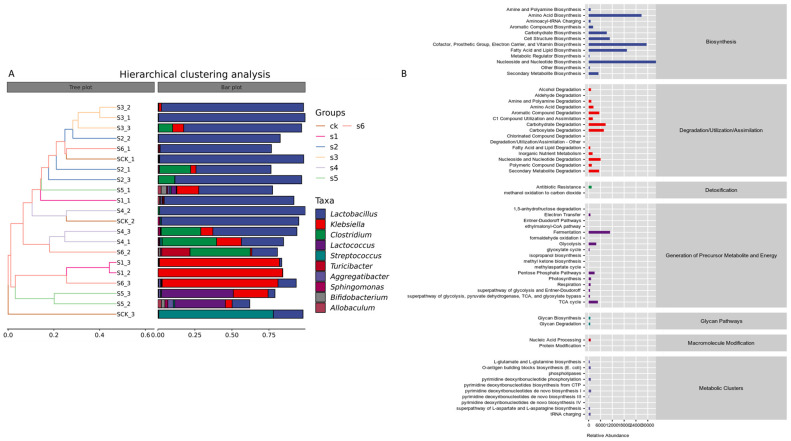
Hierarchical cluster analysis and metabolic pathway statistics. (**A**) In the hierarchical cluster figure, the left side panel is a hierarchical clustering tree, where the samples are clustered based on their resemblance, and the smaller the branch length among the samples, the more identical the two samples are. On the right panel (plotted by default) is a stacked histogram of the 10 most prevalent genera. CK: control. S1: WT Kp138. S2: ∆Kp138. S3: WT KpC4. S4: ∆KpC4. S5: WT KpE4. S6: ∆KpE4. (**B**) *Klebsiella pneumoniae* impacts metabolic pathways within gut bacterial communities. Functional secondary classifications from the KEGG database are categorized at the genus level. The lowest column in the table is the abundance or count of the first-level pathway/classification, the second column is the second-level pathway/classification, and the third column is the functional pathway/classification.

To shed light on microorganisms roles in the gut of experimental mice groups and to distinguish genera across groups, PPICRUSt2 (system genetic investigation of communities by reconstruction of unobserved states) was utilized for predicting the metabolic activity of the microbial community on the KEGG. (https://www.kegg.jp/) and MetaCyc (https://metac.yc.org/) databases. Differential metabolic pathways across the groups were screened based on logFC and adj *p* values, and the species makeup of metabolic pathways was examined (Figure 7A). Most bacterial taxa were functionally linked to precursor metabolite, degradation/utilization/assimilation, biosynthesis, energy production, and the establishment of metabolic clusters.

Within the biosynthesis category, nucleoside and nucleotide synthesis were most abundant (35,815.31%). Other biosynthetic categories include amino acid (26,836.14%); lipid and fatty acid (19,413%); carbohydrate (9201.28%); cell structure (10,743.36%); secondary metabolite (4990.92%); aromatic compound (2260.35%); other compound (621.22%); and the biosynthesis of amine and polyamine (1043.42%), aminoacyl-tRNA charging (995.83%), and metabolic regulator biosynthesis (426.17%). Within the degradation/utilization/assimilation category, the most abundant process was found in the degradation of carbohydrates (8605.62%). This was followed by carboxylate degradation (7705.94%), nucleoside and nucleotide (6077.32%), aromatic compounds (5411.78%), secondary metabolite (5334.73%), and amino acid (2436.08%) degradation; C1 compound assimilation and utilization (2116.49%); inorganic nutrient metabolism (1985.95%); amine and polyamine (1360.67%), polymeric compound (1587.09%), alcohol degradation (1199.8%), and fatty acid and lipid degradation (827.98%) (Figure 7B).

## 3. Discussion

Culturing and traditional isolation techniques have yielded limited insights into *Klebsiella* infection in the mice gut. In this study, we described the microbial community in healthy, wild, and mutant *Kp*-treated mice. We used Illumina sequencing to describe the changes in microbial communities during infection under different bacterial strains and host immune response against disease. Firmicutes were the top abundant bacterial phylum in the gut of healthy, wild, and Mutant-treated mice except for clinical wild strain Kp138.

The results align with the earlier research on the microbiota in the liver [18]. The *rpoS* mutation in the present study significantly impacted the bacterial diversity index in mice. According to Chao1, Simpson, observed-species, and Shannon values, the virulence of distinct *Klebsiella* strains was accompanied by variations in the microbial community richness. The gut microbiota is crucial for preserving human health, and new research indicates a link between intestinal microbial dysbiosis and long-term illnesses, including chronic hepatitis and diabetes [19,20,21]. Furthermore, the gut microbiota is thought to be an extended host defense mechanism that strengthens the body defenses against pathogen invasion and disease. The mechanism controlling the dynamic interactions between pathogenic bacteria and microbiota is yet fragmented. Nevertheless, in the current investigation, we explained how healthy mice’s natural microbiota was affected by the opportunistic pathogen *K. pneumoniae* and its variants. Our results suggest that the *K. pneumoniae* WT Kp138, WT KpE4, and WT KpC4 isolates can significantly alter the microbiota composition in healthy mice, leading to gut abnormalities. Through comparative analysis of the microbiota compositions between healthy and infected mice, we also found evidence that the alterations in particular bacterial species in the gut correlate with infection.

We discovered that inoculation with *K. pneumoniae* by oral gavage (Kp138, KpE4, KpC4, and their respective mutants) altered the composition of gut microbiota in both mice groups that were infected with wild-type and mutant, respectively, compared to the healthy mouse group. Still, a direct comparison of the gut microbiota of WT and Mutant-treated mice revealed particular changes linked to the gut infection. Most notably, the gut infection was linked to a significant reduction in the *Lactobacillus* spp., an abundance that is a crucial part of the gut microbiota and a member of the *Lactobacillales* within the *Bacilli* class of Firmicutes [22]. Numerous investigations have further shown the vital role of *Lactobacillus* in host health. Some strains, such as *Lactobacillus rhamnosus*, have been isolated and used as probiotics [23,24]. We speculate that the significant decline in *Lactobacillus* abundance may be closely associated with a gut infection caused by *Klebsiella* due to the critical function that *Lactobacillus* plays. It has been shown that enteropathogenic bacteria, such as *Salmonella typhimurium* and enterotoxigenic *Escherichia coli*, are less virulent when pro-biotic *Lactobacilli* are present [25,26,27]. In our study, wild-type *K. pneumoniae*-treated mice exhibited higher disease infection and were accompanied by a decreased abundance of *Lactobacillus*.

Conversely, mice treated with the mutant *K. pneumoniae* did not develop severe *Klebsiella* infection or show a reduction in *Lactobacillus*. Healthy mice gut possessed a higher abundance of *Lactobacillus* and a small fraction of Protobacteria, which proved that *Klebsiella* is present harmlessly in the mice gut and plants as an endophyte https://medicalxpress.com/news/2024-03-klebsiella-pneumoniae-opportunistic-pathogen-harmless.html [28]. When *K. pneumoniae* isolates produce a gut infection, *Lactobacillus* may also be a decisive factor in disease outcome. However, aside from *Lactobacillus*, *K. pneumoniae* isolates-induced changes in microbiota composition may play a role in the pathogenicity of *Kp* infection.

The study offers novel insights into the microbial community structure, providing a detailed exploration of bacterial diversity and abundance in the gut during *Klebsiella* infection. It lays a theoretical foundation for developing innovative disease management strategies. Using Illumina-based sequencing, our research signifies the initial characterization of bacterial diversity, abundance, and functional predictions in the gut microbiota of healthy, wild-type and mutant *Kp*-treated mice. Significant variations in alpha diversity indices were recorded across all mouse groups under investigation. Further experiments are warranted to elucidate the specific functions of different bacteria in the gut microbiome. In summary, our research shows that a *K. pneumoniae* gut infection causes alterations in the gut microbiota in healthy mice and, most notably, a decrease in *Lactobacillus*. This suggests that alteration in microbiota compositions is an essential pathogenic mechanism underlying gut infection.

## 4. Materials and Methods

### 4.1. Ethical Statement

The Animal Care and Use Committee of Yunnan Agricultural University (GB14925–2010), Kunming, China, approved all of the animal experiments.

### 4.2. Mice Collection

Kunming mice (*Mus musculus* albus), aged 6 to 8 weeks and weighing 20 ± 2 g, were acquired from Kunming Medical University, Kunming, China. The management of the mice was conducted in strict adherence to the guidelines outlined in the “Experimental Animal Environment and Facilities” (National Standard GB 14925-2010) and Yunnan Provincial Regulations on the Administration of Laboratory Animals (No. 59). The experiment was carried out after a 3–4 days acclimation period following the mice purchase. The mice were provided with food and water and were placed individually in aerated cages upon their transfer to the Biosafety Level 2 laboratory, where the infectious experiments were conducted.

### 4.3. Experimental Setup

This study was conducted at the State Key Laboratory for Conservation and Utilization of Bioresources in Yunnan, Kunming, China. Three wild-type (WT) strains of *K. pneumoniae* (Kp138 from humans, KpC4 from maize, and KpE4 from ditch water environment) were isolated and stored at −80 °C in the “State Key Laboratory for Conservation & Utilization of Bioresources in Yunnan Agricultural University, Kunming, China.

Mutants of all three strains were constructed using the allelic-exchange method, where homologous recombination integrated the suicide plasmid psr47S containing a 1.5-kbp fragment of the *rpoS* gene into the chromosomal *rpoS* gene of *K. pneumoniae* through conjugation. This results in a single-crossover mutant with antibiotic resistance. A counter-selection process using sucrose eliminates the vector’s selective markers, forming unmarked double-crossover mutants. This two-step process produces seamless mutations with precision to a single DNA base pair [29]. Before the experiment, 100 μL samples from every six bacterial strains were taken from the preserved sample at −80 °C and cultivated in LB broth at 37 °C with 180 rpm for 24 h. A spectrophotometer was used to monitor the cell growth, and suspension of six bacteria strains was adjusted to a concentration of 1 × 10^8^ CFU/mL, which was then cultured in 5 mL of Luria Bertani (LB) medium. A total of 21 mice were used in this experiment, and seven treatments, including a control, were applied. Three mice were used for each treatment (*n* = 3). Mice were dissected on day 13 post-infection [30].

### 4.4. Gut-Colonization Model

In the first step of the experiment, the mice were administered a 100 μL dose of (*K. pneumoniae*) wild-type and mutant inoculum via oral gavage slowly to avoid choking. To detect the signs of distress, animals were routinely checked for their weight and other visible signs. The mice infected with the pathogen exhibited different moderate to severe symptoms of distress, such as hunched body posture, separation from the group, inactivity, fur piloerection, asthma, and weight loss, which were recorded during the experiment. The mice guts were carefully incised and examined visually to assess any damage.

### 4.5. Microbiome Analysis in the Presence of Wild-Type and Mutant Strain

The incised gut was carefully collected, washed with ddH_2_O twice, kept in sterile zipper bags, and stored at −80 °C. The OMEGA Soil DNA Kit (M5635-02) from Omega Bio-Tek (Norcross, GA, USA) was used to extract genomic DNA from all samples according to the manufacturer’s instructions. The extracted DNA samples were kept at −20 °C until further procedure. The concentration and purity of the extracted DNA were determined using a NanoDrop NC2000 spectrophotometer from Thermo Fisher Scientific (Waltham, MA, USA), and the DNA’s quality was evaluated using agarose gel electrophoresis. The libraries were produced in batches, measured, and sequenced on an Illumina Platform using a 500 bp read length (2 × 250 bp). Amplicon identification and quantification evaluations were analyzed using R packages (v3.2.0). Initially, sequence quality was assessed using FASTQC software (v.0.11.9) (https://www.bioinformatics.babraham.ac.uk/projects/fastqc/ accessed on 19 July 2023). Subsequently, DADA2 [31] was employed to generate amplicon sequence variants (ASVs). Briefly, sequences underwent visual inspection before applying filtering and trimming functions, followed by error modeling for denoising and merging the reads using default parameters. 

### 4.6. 16S rRNA Gene Amplicon Sequencing

The V3-V4 region of the bacterial 16S rRNA genes was amplified by PCR using the forward primer 338F (5′-ACTCCTACGGGAGGCAGCA-3′) and reverse primer 806R (5′-GGACTACHVGGGTWTCTAAT-3′). The primers were modified with sample-specific 7 bp barcodes to facilitate multiplex sequencing. The PCR mixture consisted of 5 μL of buffer, 2 μL of dNTPs (2.5 mM), 0.25 μL of Fast pfu DNA Polymerase (5 U/μL), 1 μL each of forward and reverse primers (10 μM), 14.75 μL of ddH_2_O, and 1 μL of DNA template. The thermal cycling technique was performed with a 5 min denaturation at 98 °C, followed by 25 cycles of denaturation for 30 s, annealing at 53 °C for 30 s, and extension at 72 °C for 45 s. The amplification was determined by a final extension step of 5 min at 72 °C. The PCR amplicons were purified using Vazyme VAHTSTM DNA Clean Beads (Vazyme, Nanjing, China) and quantified with the Quant-iT PicoGreen dsDNA Assay Kit (Invitrogen, Carlsbad, CA, USA). Following individual measurements, the amplicons were pooled in equimolar amounts. Pair-end sequencing with a read length of 2 × 250 bp was performed using the Illumina NovaSeq technology and the NovaSeq 6000 SP Reagent Kit (500 cycles) at Shanghai Personal Biotechnology Co., Ltd, Shanghai, China.

### 4.7. Sequence Analysis

Microbiome bioinformatics analyses were carried out using QIIME2 2022.11 [32], with minor alterations based on the official tutorials at https://docs.qiime2.org/2022.11/tutorials/. In brief, the raw sequence data underwent demultiplexing using the Demux plugin, followed by primer trimming using the Cutadapt plugin [33]. Sequences were then quality filtered, combined, denoised, and chimeras eliminated using the DADA2 plugin [31]. Amplicon sequence variations (ASVs) that were nonsingletons were aligned using MAFFT [34] and used to create a phylogenetic tree using FastTree2 [35].

### 4.8. Taxonomic Composition Analysis

The classify-sklearn naive Bayes taxonomy classifier within the feature-classifier plugin [36] was used to classify ASVs, employing the database provided by [37]. We analyzed the 10–20 most abundant taxa from each group using comprehensive data.

### 4.9. Alpha and Beta Diversity Index Analysis

Alpha-diversity metrics Chao1 [38], Shannon [39,40], Observed species, Faith’s PD [41], Simpson [42], Good’s coverage [43] and Pielou’s evenness [44], and beta diversity metrics (weighted UniFrac [45], Jaccard distance, Bray–Curtis dissimilarity, and unweighted UniFrac[46]), were calculated using the diversity plugin. Samples were rarefied to an equal number of sequences per sample.

QIIME2 and the R package (v3.2.0) were mainly used to analyze sequence information. QIIME2 generated ASV-level alpha diversity metrics such as the Chao1 richness estimator, Shannon diversity index, observed species, Faith’s PD, Pielou’s evenness, Simpson index, and Good’s coverage. These metrics were represented using box plots. In addition, sorted abundance curves at the ASV level were used to cR pRompare the evenness and richness of the samples. Beta diversity analysis investigated fundamental differences in bacterial communities within samples using Jaccard distance metrics [47], UniFrac distance metrics [45,46], and Bray–Curtis dissimilarity metrics [48]. These metrics were shown using principal coordinate analysis (PCoA) and hierarchical clustering with the unweighted pair-group approach with arithmetic means (UPGMA) [49]. In addition, principal component analysis (PCA) was performed using genus-level compositional profiles.

### 4.10. Analysis of Differentiation among Groupings at Each Taxonomic Level

PERMANOVA (permutational multivariate analysis of variance) [50], Permdisp [51], and ANOSIM (analysis of similarities) [52,53] were used with QIIME2 to determine the importance of variations in microbial community framework between groups. A Venn diagram was created utilizing the R package “VennDiagram” to depict shared and unique ASVs among samples or groups based on ASV occurrence, regardless of relative abundance [54]. In addition, LEfSe (linear discriminant analysis effect size) was used with default parameters to find differentially abundant taxa across groups [55].

### 4.11. Construction of Association Network and Prediction of Microbial Metabolic Functions

A co-occurrence association network analysis was conducted using SparCC analysis, with a pseudo-count value set to 10^6^. Utilizing random matrix theory-based approaches implemented in the R package RMThreshold, a correlation coefficient threshold of 70 was established. Employing the correlation coefficients, we built a co-occurrence network with nodes representing ASVs and edges representing correlations between these ASVs. The network was displayed using the R tools graph. Microbial function predictions were performed using PICRUSt2 (Phylogenetic Investigation of Communities by Re-construction of Unobserved States) [56], which used data from the MetaCyc (https://metacyc.org/) and KEGG (https://www.kegg.jp/) databases. Based on the projected results, different metabolic pathways across groups were discovered using adjusted *p* values, species composition analysis of metabolic pathways, and log fold changes (logFC) to predict the principal coordinate analysis (PCoA) at the operational level of the operational unit.

## Data Availability

The raw sequence data obtained in this study were deposited in the Sequence Read Archive (SRA) of the National Center for Biotechnology Information (NCBI) under accession number PRJNA1114401. All authors approved the disclosure of data because this study did not involve human subjects.

## References

[1] Ward N.L., Pieretti A., Dowd S.E., Cox S.B., Goldstein A.M. (2012). Intestinal aganglionosis is associated with early and sustained disruption of the colonic microbiome. Neurogastroenterol. Motil..

[2] Kang D.-W., Park J.G., Ilhan Z.E., Wallstrom G., LaBaer J., Adams J.B., Krajmalnik-Brown R. (2013). Reduced incidence of *Prevotella* and other fermenters in intestinal microflora of autistic children. PLoS ONE.

[3] Pascoe E.L., Hauffe H.C., Marchesi J.R., E Perkins S. (2017). Network analysis of gut microbiota literature: An overview of the research landscape in non-human animal studies. ISME J..

[4] Liu Y., Huang L., Cai J., Zhu H., Li J., Yu Y., Xu Y., Shi G., Feng Y. (2023). Clinical characteristics of respiratory tract infection caused by *Klebsiella pneumoniae* in immunocompromised patients: A retrospective cohort study. Front. Cell. Infect. Microbiol..

[5] Guo Y., Wang S., Zhan L., Jin Y., Duan J., Hao Z., Lv J., Qi X., Chen L., Kreiswirth B.N. (2017). Microbiological and clinical characteristics of hypermucoviscous *Klebsiella pneumoniae* isolates associated with invasive infections in China. Front. Cell. Infect. Microbiol..

[6] Lee C.-R., Lee J.H., Park K.S., Jeon J.H., Kim Y.B., Cha C.-J., Jeong B.C., Lee S.H. (2017). Antimicrobial resistance of hypervirulent *Klebsiella pneumoniae*: Epidemiology, hypervirulence-associated determinants, and resistance mechanisms. Front. Cell. Infect. Microbiol..

[7] Alexander A.D., Orcutt R.P., Henry J.C., Baker J., Bissahoyo A.C., Threadgill D.W. (2006). Quantitative PCR assays for mouse enteric flora reveal strain-dependent differences in composition that are influenced by the microenvironment. Mamm. Genome.

[8] Bendtsen K.M.B., Krych L., Sørensen D.B., Pang W., Nielsen D.S., Josefsen K., Hansen L.H., Sørensen S.J., Hansen A.K. (2012). Gut microbiota composition is correlated to grid floor induced stress and behavior in the BALB/c mouse. PLoS ONE.

[9] Dong T., Joyce C., Schellhorn H.E. (2008). The role of RpoS in bacterial adaptation. Bacterial Physiology: A Molecular Approach.

[10] Hengge-Aronis R.J.M. (2002). Signal transduction and regulatory mechanisms involved in control of the σS (RpoS) subunit of RNA polymerase. Microbiol. Mol. Biol. Rev..

[11] Fang F.C., Libby S.J., A Buchmeier N., Loewen P.C., Switala J., Harwood J., Guiney D.G. (1992). The alternative sigma factor katF (rpoS) regulates *Salmonella virulence*. Proc. Natl. Acad. Sci. USA.

[12] Yildiz F.H., Schoolnik G.K. (1998). Role of rpoS in stress survival and virulence of *Vibrio cholerae*. J. Bacteriol..

[13] Suh S.-J., Silo-Suh L., Woods D.E., Hassett D.J., West S.E.H., Ohman D.E. (1999). Effect of *rpoS* mutation on the stress response and expression of virulence factors in *Pseudomonas aeruginosa*. J. Bacteriol..

[14] Wang Y., Kim K.S. (2000). Effect of *rpoS* mutations on stress-resistance and invasion of brain microvascular endothelial cells in *Escherichia coli* K1. FEMS Microbiol. Lett..

[15] Battesti A., Majdalani N., Gottesman S. (2011). The *rpoS*-mediated general stress response in *Escherichia coli*. Annu. Rev. Microbiol..

[16] Dong T., Schellhorn H.E. (2010). Role of RpoS in virulence of pathogens. Infect. Immun..

[17] Price S.B., Cheng C.-M., Kaspar C.W., Wright J.C., DeGraves F.J., Penfound T.A., Castanie-Cornet M.-P., Foster J.W. (2000). Role of *rpoS* in acid resistance and fecal shedding of *Escherichia coli* O157:H7. Appl. Environ. Microbiol..

[18] Hmelo L.R., Borlee B.R., Almblad H., E Love M., E Randall T., Tseng B.S., Lin C., Irie Y., Storek K.M., Yang J.J. (2015). Precision-engineering the *Pseudomonas aeruginosa* genome with two-step allelic exchange. Nat. Protoc..

[19] Dong T., Coombes B.K., Schellhorn H.E. (2009). Role of RpoS in the virulence of *Citrobacter rodentium*. Infect. Immun..

[20] Callahan B.J., Mcmurdie P.J., Rosen M.J., Han A.W., Johnson A.J.A., Holmes S.P. (2016). DADA_2_: High-resolution sample inference from Illumina amplicon data. Nat. Methods.

[21] Bolyen E., Rideout J.R., Dillon M.R., Bokulich N.A., Abnet C., Al-Ghalith G.A., Alexander H., Alm E.J., Arumugam M., Asnicar F. (2018). QIIME 2: Reproducible, interactive, scalable, and extensible microbiome data science. PeerJ Prepr..

[22] Martin M. (2011). Cutadapt removes adapter sequences from high-throughput sequencing reads. EMBnet. J..

[23] Katoh K., Misawa K., Kuma K.i., Miyata T. (2002). MAFFT: A novel method for rapid multiple sequence alignment based on fast Fourier transform. Nucleic Acids Res..

[24] Price M.N., Dehal P.S., Arkin A.P. (2009). FastTree: Computing large minimum evolution trees with profiles instead of a distance matrix. Mol. Biol. Evol..

[25] Bokulich N.A., Kaehler B.D., Rideout J.R., Dillon M., Bolyen E., Knight R., Huttley G.A., Gregory Caporaso J. (2018). Optimizing taxonomic classification of marker-gene amplicon sequences with QIIME 2’s q2-feature-classifier plugin. Microbiome.

[26] Kõljalg U., Nilsson R.H., Abarenkov K., Tedersoo L., Taylor A.F.S., Bahram M., Bates S.T., Bruns T.D., Bengtsson-Palme J., Callaghan T.M. (2013). Towards a unified paradigm for sequence-based identification of fungi. Mol. Ecol..

[27] Chao A. (1984). Nonparametric estimation of the number of classes in a population. Scand. J. Stat..

[28] Shannon C.E. (1948). A mathematical theory of communication. Bell Syst. Tech. J..

[29] Shannon C.E. (2001). A mathematical theory of communication. ACM SIGMOBILE Mob. Comput. Commun. Rev..

[30] Faith D.P. (1992). Conservation evaluation and phylogenetic diversity. Biol. Conserv..

[31] Simpson E.H. (1949). Measurement of diversity. Nature.

[32] Good I.J. (1953). The population frequencies of species and the estimation of population parameters. Biometrika.

[33] Pielou E.C. (1966). The measurement of diversity in different types of biological collections. J. Theor. Biol..

[34] Lozupone C.A., Hamady M., Kelley S.T., Knight R. (2007). Quantitative and qualitative β diversity measures lead to different insights into factors that structure microbial communities. Appl. Environ. Microbiol..

[35] Lozupone C., Knight R. (2005). UniFrac: A new phylogenetic method for comparing microbial communities. Appl. Environ. Microbiol..

[36] Jaccard P. (1908). Nouvelles recherches sur la distribution florale. Bull. Soc. Vaud. Sci. Nat..

[37] Bray J.R., Curtis J.T. (1957). An ordination of the upland forest communities of southern Wisconsin. Ecol. Monogr..

[38] Ramette A. (2007). Multivariate analyses in microbial ecology. FEMS Microbiol. Ecol..

[39] McArdle B.H., Anderson M.J. (2001). Fitting multivariate models to community data: A comment on distance-based redundancy analysis. Ecology.

[40] Anderson M.J., Ellingsen K.E., McArdle B.H. (2006). Multivariate dispersion as a measure of beta diversity. Ecol. Lett..

[41] Clarke K.R. (1993). Non-parametric multivariate analyses of changes in community structure. Aust. J. Ecol..

[42] Warton D.I., Wright S.T., Wang Y. (2012). Distance-based multivariate analyses confound location and dispersion effects. Methods Ecol. Evol..

[43] Zaura E., Keijser B.J., Huse S.M., Crielaard W. (2009). Defining the healthy “core microbiome” of oral microbial communities. BMC Microbiol..

[44] Segata N., Izard J., Waldron L., Gevers D., Miropolsky L., Garrett W.S., Huttenhower C. (2011). Metagenomic biomarker discovery and explanation. Genome Biol..

[45] Douglas G.M., Maffei V.J., Zaneveld J., Yurgel S.N., Brown J.R., Taylor C.M., Huttenhower C., Langille M.G.J.B. (2019). PICRUSt2: An improved and extensible approach for metagenome inference. bioRxiv.

[46] Chen N., Ling Z.-X., Jin T.-T., Li M., Zhao S., Zheng L.-S., Xi X., Wang L.-L., Chen Y.-Y., Shen Y.-L. (2018). Altered profiles of gut microbiota in *Klebsiella pneumoniae*-induced pyogenic liver abscess. Curr. Microbiol..

[47] Lynch S.V., Pedersen O. (2016). The human intestinal microbiome in health and disease. N. Engl. J. Med..

[48] Roderburg C., Luedde T. (2014). The role of the gut microbiome in the development and progression of liver cirrhosis and hepatocellular carcinoma. Gut Microbes.

[49] Sabatino A., Regolisti G., Cosola C., Gesualdo L., Fiaccadori E. (2017). Intestinal microbiota in type 2 diabetes and chronic kidney disease. Curr. Diabetes Rep..

[50] Claesson M.J., van Sinderen D., O’Toole P.W. (2008). *Lactobacillus* phylogenomics–towards a reclassification of the genus. Int. J. Syst. Evol. Microbiol..

[51] Salminen M.K., Tynkkynen S., Rautelin H., Saxelin M., Vaara M., Ruutu P., Sarna S., Valtonen V., Järvinen A. (2002). *Lactobacillus* bacteremia during a rapid increase in probiotic use of *Lactobacillus rhamnosus* GG in Finland. Clin. Infect. Dis..

[52] Sullivan Å., Erik Nord C. (2006). Probiotic lactobacilli and bacteraemia in Stockholm. Scand. J. Infect. Dis..

[53] Bernet-Camard M.F., Liévin V., Brassart D., Neeser J.R., Servin A.L., Hudault S. (1997). The human Lactobacillus acidophilus strain LA1 secretes a nonbacteriocin antibacterial substance(s) active in vitro and in vivo. Appl. Environ. Microbiol..

[54] Gopal P.K., Prasad J., Smart J., Gill H.S. (2001). In vitro adherence properties of *Lactobacillus rhamnosus* DR20 and *Bifidobacterium lactis* DR10 strains and their antagonistic activity against an enterotoxigenic *Escherichia coli*. Int. J. Food Microbiol..

[55] Hudault S., Liévin V., Bernet-Camard M.F., Servin A.L. (1997). Antagonistic activity exerted in vitro and in vivo by *Lactobacillus casei* (strain GG) against *Salmonella typhimurium* C5 infection. Appl. Environ. Microbiol..

[56] Reyna-Flores F., Barrios-Camacho H., Dantán-González E., Ramírez-Trujillo J.A., Lozano Aguirre Beltrán L.F., Rodríguez-Medina N., Garza-Ramos U., Suárez-Rodríguez R. (2018). Draft genome sequences of endophytic isolates of *Klebsiella variicola* and *Klebsiella pneumoniae* obtained from the same sugarcane plant. Genome Announc..

